# A Muscle Variant of the Lateral Neck Region: The Cleido-Vertebral Muscle

**DOI:** 10.22038/IJORL.2023.70325.3388

**Published:** 2023-05

**Authors:** Daniela Parrino, Enzo Emanuelli

**Affiliations:** 1 *Department of Otorhinolaryngology Head and Neck Surgery, ASST Sette Laghi, Ospedale di Circolo e Fondazione Macchi, Varese, Italy. *; 2 *Unit of Otorhinolaryngology, AULSS 2-Marca Trevigiana, Treviso, Italy.*

**Keywords:** Anomalous muscle, Neck muscle variant, Neck dissection, Omohyoid

## Abstract

**Introduction:**

Anatomical variations of the neck muscles have previously been reported, involving in particular the omohyoid and sternothyroid muscles. We herein report a novel variant neck muscle found during a routine surgical procedure.

**Case Report:**

A 63-year-old women underwent a pelvi-mandibulectomy with bilateral neck dissection for a squamous cell carcinoma of the floor of mouth pT3N1. On the right neck dissection, the present peculiar muscle was discovered. It was located in the lateral region of the neck, deeply to the sternocleidomastoid muscle and caudally to the hyoid bone. It took origin from the sixth cervical vertebrae’s transverse process and attached caudally to the middle third of the clavicular bone, after having passed superficially to the omohyoid muscle’s intermediate tendon.

**Conclusions:**

Neck muscles are important during head and neck surgery due to their significance as surgical landmarks and their relationship with noble vessels. Being aware of possible variant that can alter classical anatomical reference points is important to prevent iatrogenic trauma.

## Introduction

Various anomalous muscles of the neck have previously been reported. The reports available in literature mostly regards findings during cadaveric dissection, and the anatomical variants described involve the omohyoid and sternothyroid muscles (1-5). An additional infrahyoid muscle, defined as cleido-hyoid, has also been depicted and its possible variants have been described (6,7). As well, during dissection studies, an abnormal neck muscle deep to the sternocleidomastoid muscle was encountered and classified as cleido-occipital muscle, according to its origin and insertion (8). 

Knowing the existence of neck muscle anatomical variants is important to head and neck surgeons, as those represent surgical landmarks during neck dissection, larynx and thyroid surgeries. 

We herein report the case of a muscle variant of the lateral region of the neck observed during a routine surgical procedure, that has never been previously described. In light of its course, we named it the cleido-vertebral muscle.

## Case Report

A 63-year-old women underwent a pelvi-mandibulectomy with bilateral neck dissection (pull through technique) for a squamous cell carcinoma of the floor of mouth pT3N1. Performing the right neck dissection, the present peculiar muscle was discovered. 

The anomalous muscle was located in the lateral region of the neck, deeply to the sternocleidomastoid muscle and caudally respect to the hyoid bone. It took origin from the sixth Cervical vertebrae’s transverse process and, passing deeply to the internal jugular vein (IJV), continued down forward and laterally in direction of the omohyoid muscle. 

**Fig 1:**
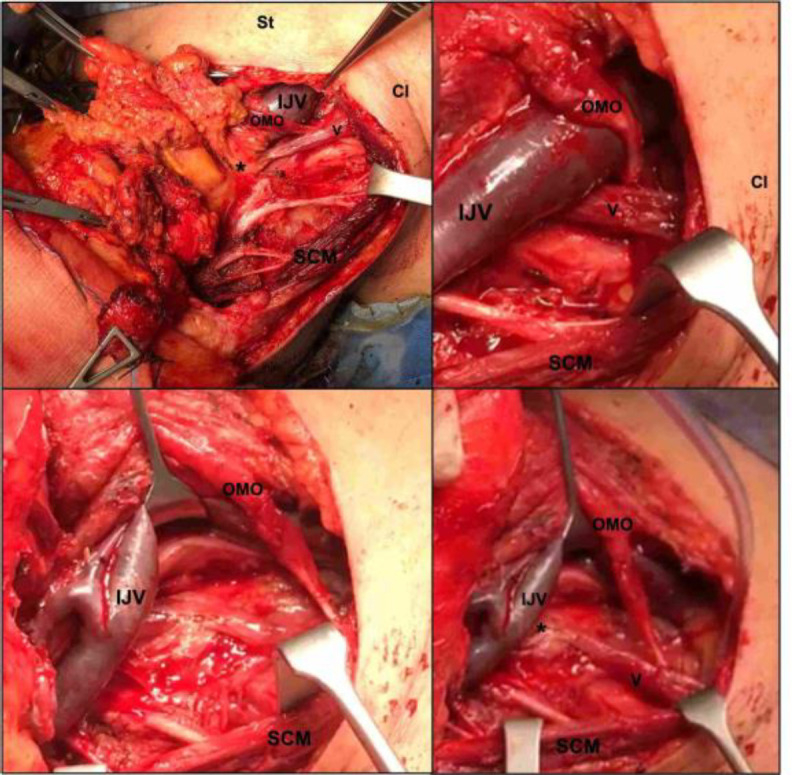
Panel showing intraoperative pictures of the cleido-vertebral muscle. Cl: clavicle; IJV: internal jugular vein; OMO: omohyoid muscle; SCM: sternocleidomastoid muscle; St: sternum; V: variant muscle; Asterisk indicate the vertebrae’s transverse process projection

Reached the omohyoid muscle, the anomalous muscle overpassed it superficial to its intermediate tendon, and then proceeded inferiorly to take insertion to the middle third of the clavicular bone, after having passed superficially to the external jugular vein (Figure 1). A branch of the ansa cervicalis innervated the muscle. 

**Fig 2:**
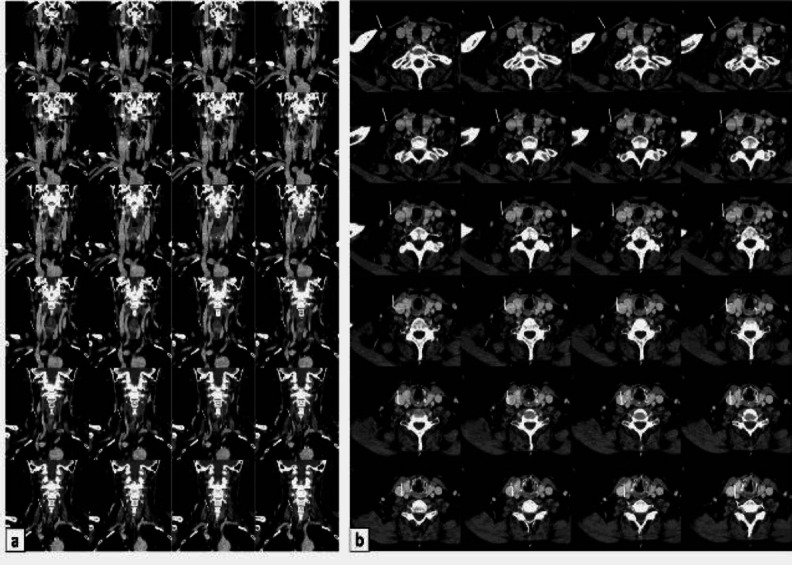
Panel showing the muscle on the contrasted enhanced CT scan. Coronal view (a) and axial view (b). White arrow indicates the cleido-vertebral muscle; asterisk the sixth cervical vertebrae’s transverse process

The preoperative contrast enhanced Computed Tomography (CT) of the neck was then accurately analyzed focusing on muscular structures, and the muscle was pinpointed both in axial and coronal sections (Figure 2a, 2b).

No anomalous structures were encountered during the contralateral neck dissection.

## Discussion

The omohyoid muscle was considered a landmark for classify lymph node levels in head and neck cancer surgery (9). Nowadays, its significance during neck dissection resides more in being a reference for identify the distal portion of the IJV and in its close relation to the brachial plexus. 

Thus, any variation of it may mislead level classification of node metastasis and condition the risk of vascular and nervous structures damage during neck surgeries (10-11). The possibility of finding omohyoid anomalies does not seem to be infrequent (2). According to the origin, the course, the insertion and the number of bellies of this muscle, Yamada3 in 1960 classified its anatomic variants in 6 types (Table 1). Being aware of these variations is paramount to head and neck surgeons in order to avoid an increased risk of IJV rupture and brachial plexus damage. 

**Table 1:** Yamada’s classification of the anomalous omohyoid muscle (3)

Type I	Absence of omohyoid muscle (whole or part) and/or intermediate tendon.
Type II	Supernumerary bundles of superior belly or duplication of whole muscle.
Type III	Abnormal pattern of inferior belly (divided into two or three fasciculi).
Type IV	Cleido-hyoid muscle or accessory cleido-hyoid muscle.
Type V	Abnormal pattern of insertion of omohyoid muscle (shortening of superior belly).
Type VI	Accessory omohyoid muscle (M. omohyoideus accessorius).
	

It is also important to know any anomalies of structures surrounding the omohyoid muscle, as they can also condition surgical landmarks and make dissection more complex. The muscle herein described, that we define cleido-vertebral muscle, during its course between the sixth cervical vertebrae’s transverse process and the clavicular bone, took relation with the omohyoid muscle, crossing over its intermediate tendon. These variations could be derived from an anomalous merge between the ventrolateral portion of the somite’s dermatomyotome involved in the development of the prevertebral neck muscles and the ‘lingual-infrahyoid-diaphragmatic band’ which forms the anterior neck muscles (10,4). 

At first sight, the cleido-vertebral caudal portion that inserts on the clavicle may be mistaken for the inferior belly of the omohyoid muscle or for one its anatomic variant (i.e. cleido-hyoid) (7). Moreover, omohyoid and sternothyroid muscles variants may have a direct effect on the lumen of the surrounding vessels and cause compression of the IJV with subsequent modification of the intracerebral venous hemodynamics (2,5). This is not the case of our muscle as it lied posteriorly to the IJV without contacting, straightening nor compressing it. 

Preoperative imaging is generally useless to detect the anatomic variation, as the radiologist primarily focuses on describing the oncological pathology characteristics. 

However, once an anomalous muscle is identified during live surgery, it can be challenging for both the surgeon and the radiologist to depict it also in preoperative imaging, to better evaluate its relationship with surrounding structures. 

A contrasted enhanced Magnetic Resonance imaging surely would have shown the present muscle event better than the CT scan, but unfortunately our patient was staged with contrasted enhanced and positron emission tomography CT scans, because she suffers from claustrophobia.

## Conclusion

The muscles of the neck are important structure for head and neck surgery due to their significance as surgical landmarks and their close relationship with noble vessels and brachial plexus. Therefore, head and neck surgeons should be aware of possible variant that can alter classical anatomical reference points used during neck dissection, in order to prevent iatrogenic trauma. 
